# Social implications arise in embodied music cognition research which can counter musicological “individualism”

**DOI:** 10.3389/fpsyg.2014.00676

**Published:** 2014-07-18

**Authors:** Nikki Moran

**Affiliations:** Reid School of Music, Edinburgh College of Art, Edinburgh UniversityEdinburgh, UK

**Keywords:** music performance, music cognition, social interaction, individualism, empirical research

## Abstract

The agenda in music research that is broadly recognized as embodied music cognition has arrived hand-in-hand with a social interpretation of music, focusing on the real-world basis of its performance, and fostering an empirical approach to musician movement regarding the communicative function and potential of those movements. However, embodied cognition emerged from traditional cognitivism, which produced a body of scientific explanation of music-theoretic concepts. The analytical object of this corpus is based on the particular imagined encounter of a listener responding to an idealized “work.” Although this problem of essentialism has been identified within mainstream musicology, the lingering effects may spill over into interdisciplinary, empirical research. This paper defines the situation according to its legacy of individualism, and offers an alternative sketch of musical activity as performance event, a model that highlights the social interaction processes at the heart of musical behavior. I describe some recent empirical work based on interaction-oriented approaches, arguing that this particular focus – on the social interaction process itself – creates a distinctive and promising agenda for further research into embodied music cognition.

## INTRODUCTION

This paper examines the social implications of embodied music cognition, drawing attention to the individualism evident in some existing work. The interdisciplinary, empirical research that typifies the corpus broadly recognizable as embodied music cognition has the potential to bear new, social perspectives on the topic of human music-making. I argue that to bring this potential to fruition, researchers should be explicitly aware of the sorts of limitations that arise through reductive characterizations of musical phenomena. More light-heartedly, I offer the incentive that a thoroughly “social” perspective on music-making provides a valuable opportunity to compare music with kissing, and in some detail – a subject to which I will return in due course.

The collective description in this paper of all research into “embodied music cognition” demands definition. Arising in response to cognitivism’s information-based, representational explanations of mental process, “embodied cognition” acknowledges the dynamic interaction between minds, bodies and their environment, focusing on the integrity of action-perception systems (Varela et al., 1991; Anderson, 2003). Under the banner of “embodiment,” various theories ally and diverge. While all center on the notion that cognition primarily serves action, and that bodily experience informs cognition, various distinctions arise (Wilson, 2002). Theories that propose a distributed system spanning minds, bodies, and environments – thus connecting embodiment with social interaction, where others’ bodies and minds are a typical constituent of the environment – tend to advocate a shift to models of direct (rather than representational) perception (Gallagher, 2005); other theories retain models of mental representation, but propose these to be grounded in simulated actions and bodily states based on specific, situated interactions with the world (Barsalou, 2008).

In music, “embodied cognition” may signify Marc Leman’s systematic approach, theorizing the human body as the mediator between the acoustic signals in the external environment, bodily and perceptual modalities, and internal musical representation and experience (Leman, 2007). Large scale research projects dealing specifically with embodiment in music research include EmcoMetecca (Leman, 2008), The Notion of Participative and Enacting Sonic Interaction (PESI) (Correia et al., 2013) and SIEMPRE (). More generally, embodiment connotes an acknowledgment of the musician’s (and the listener’s) body in both the analysis (Iyer, 2002; Clayton et al., 2004; Toiviainen et al., 2010) and theorization (Clarke, 1993; Cox, 2001; Reybrouck, 2005; Doǧantan-Dack, 2006; Gritten and King, 2006, 2011; Rahaim, 2012; Moran, 2013a) of music performance. In this paper, I refer to embodiment in this more open sense, as a useful way of drawing together a diverse body of empirical and theoretical work.

Research that assumes musical behavior to be embodied encompasses various topics, and – when used as a broad descriptor for a swathe of current approaches – helps individual strands of work to cohere. For example, the description can be applied to programs of work in interactive performance analysis systems (Cadoz and Wanderley, 2000; Camurri et al., 2000; Goebl et al., 2005) and music and gesture research (Camurri et al., 2004; Gritten and King, 2006; Godøy and Leman, 2010; Toiviainen et al., 2010). The notion of embodiment is also fundamental to current directions in music and emotion research (Becker, 2004; Vines et al., 2005; Juslin and Timmers, 2010), and it provides a touchstone for otherwise disparate empirical studies in music performance and musical communication (Davidson, 1993; Boone and Cunningham, 2001; Iyer, 2002; Bowman, 2004; Ginsborg et al., 2004; Eitan and Granot, 2006; Timmers, 2007; Broughton and Stevens, 2009; Keller and Appel, 2010; Moran, 2013b; Kawase, 2014).

In the main, the embodiment agenda in music research has fostered an empirical approach to musician movement that attempts to account in some way for the communicative function and potential of those movements. Some of the earliest music-related publications to refer explicitly to embodiment focused on the relationship of movement and physicality to the social context of musical performance, development and cognition. For example, Davidson (2001, p. 235) drew specific attention to “the matter of how both musical and extra-musical concerns are coordinated between performer, co-performers and audience using body movements.” Iyer (2002, p. 388) pointed out how “physical embodiment and sociocultural situatedness [have a role to play] in music perception and cognition,” while Cross stated that “Music’s embodied characteristics may provide the basis for music’s capacities to coordinate and entrain action in time […] Music is embedded in social action, deriving meaning from that action and in turn endowing it with significance” (Cross, 2003, p. 108).

Focusing on the real-world basis of performance, music research dealing with embodiment has tended to bring to the foreground a more social interpretation of musical phenomena; indeed the evocation of physical presence – of movement, of outward-tending behavior – immediately calls forth a sense of communication – of communality, of expressivity, and of sharing. Thus, the topics of music performance research and of musical communication are deeply knitted together, both informing trends in musicology during the past two decades. Such attention to the corporeal aspects of performed musical phenomena should, it would seem, provide an opportunity to look right at the social event of performance as it is played out by two or more co-present, interacting human bodies. But we should ask how much this increased attention to the performing body has yet told us about the experience of music as a specifically social phenomenon; or whether we have simply scaled up our nuanced and detailed accounts of the individual, problem-solving musical mind?

I referred earlier to the idea that embodied cognition challenges traditional cognitivism, which ultimately seeks to explain mental process at an abstract, computational level. Within music, cognitive studies exemplified in the pioneering work of such music psychologists as Shepard (1982), Bharucha and Krumhansl (1983), Lerdahl and Jackendoff (1983), Deutsch (1999) and many others besides, have typically focused on the perception and analysis of acoustic signals and their communication of particular structures. Cognitive music psychology inherited its subject matter – though not without debate (Krumhansl, 1995; Cross, 1998) – from music theory. Predicated on those compositional features that are the historical subjects of classical Western European music analysis such as pitch, meter and harmony, it is important to recognize that the point of departure is a particular understanding of a specific type of musical encounter: the one between a listener and an idealized “work.” The central categories that music theoretic scholarship uses to explore this encounter do not necessarily make for the best description, or the most revealing analyses, of non-notated or oral musical traditions. As Nicholas Cook’s (1994) charge of “theorism” proposes, they may not even provide comprehensive tools for the perceptual analysis of classical Western music repertory. While the idealized “work” is a key epistemological component of one type of music theory, it does not appear to be a universal or defining category of all musical encounters, especially not when considered in cross-cultural context.

## MUSICAL ENCOUNTERS

**FIGURE 1:**
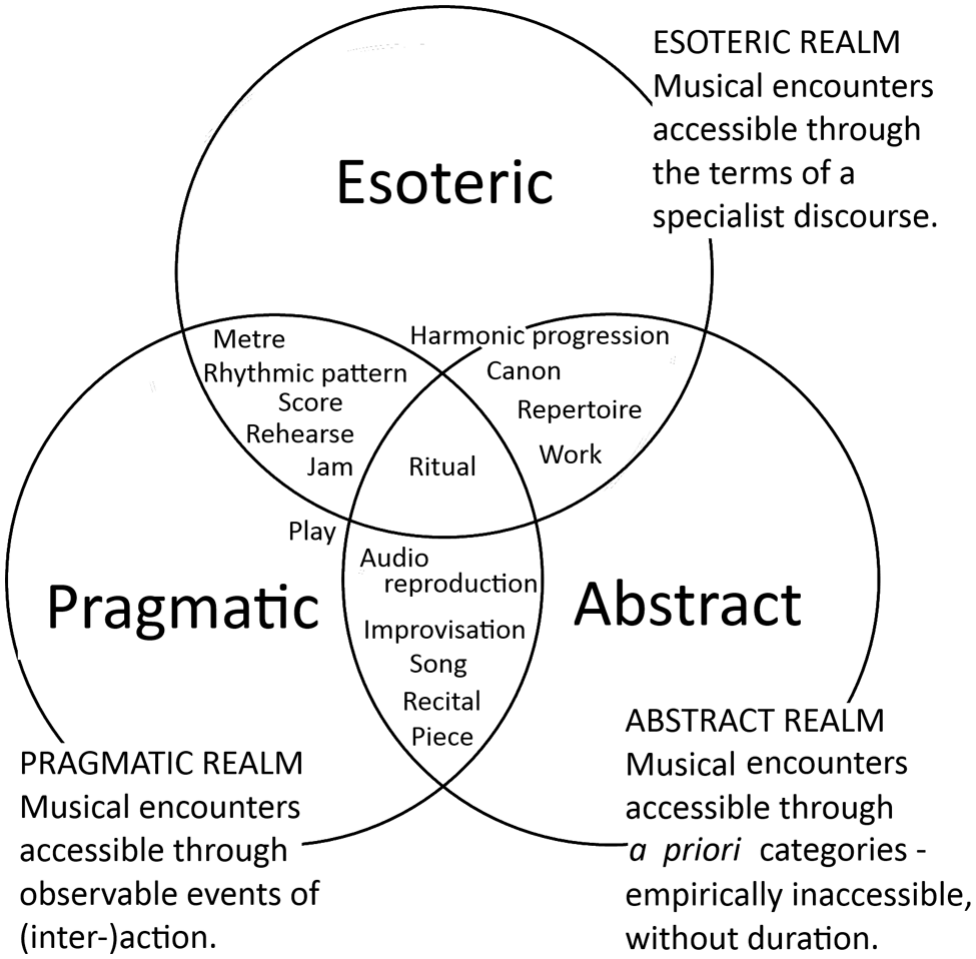
**Musical encounters and their experiential realms: pragmatic, esoteric, and abstract**.

The term “encounters” is deployed here with a particular intention to provide some purchase to the question: When we study music, what exactly is the focus of our analysis? In experience, music may be encountered in many ways. Some suggestions include: musical work; musical score; musical piece; song; performance; harmonic progression; rhythmic patterning; sonic texture; recital; rehearsal; improvisation; concert; jam; session; audio reproduction; event; play; canon; repertoire; composition; ritual; and so on. Any one of these aspects of musical experience may serve as a focus of analysis, and yet – by simply evoking these encounters in our imagination – it is clear that they are not equal objects and cannot be handled the same way as one another in analysis. Self-evidently, there is not one essential musical experience. The multiple aspects relate to very different realms of experience which are accessible through distinct ways of knowing and communicating. Access to some aspects require specialist, esoteric discourse – for example, the labeling and interpretation of harmonic progression (**Figure 1**, “Esoteric Realm”). Some encounters are observable, making them empirically accessible as events of musical action, such as rehearsing, jamming, or playing (**Figure 1**, “Pragmatic Realm”). Finally, some aspects can only be encountered as a priori categories, such as the concepts of work, canon, and repertoire. These are abstractions which cannot be observed, but which are rather contemplated and discussed (**Figure 1**, “Abstract Realm”).

The selection and configuration shown in **Figure 1** is not intended as a definitive model of musical experience, but as a precursor to research design. Given the diversity of all facets of musical experience, in order to do any sort of research in music it is necessary to specify a musical object of enquiry. For example, consider the distinction between musical work and musical play. “Play” describes a musical encounter that can be accessed by those who witness its doing [its event of (inter-)action], while “work” is inaccessible through that same pragmatic realm. It is an abstract concept whose definition also requires specialist knowledge.

The aspect of “music” taken to be the object of enquiry varies, quite naturally, depending on who is doing the choosing. Historical musicologists are more likely to be concerned with stylistic definitions and the emergence of canons than are social psychologists, who may wish to examine, say, skill acquisition, or musical identity. Popular musicologists may discuss the emergence of genre or the effect of a particular technology on compositional features of songs. The decision to focus on a particular object of musical analysis is therefore strongly influenced by the context – historical, philosophical and cultural – of the scholarly endeavor. Furthermore, we have seen that there is not one set of attributes that we can or should assume to exist for all musical phenomena. And yet precisely this problem has been documented in music scholarship, described by Philip Bohlman as the essentialization of music itself (Bohlman, 1993). The issue has also been tackled in the work of Goehr (1992) and Born (2010).

Within the bounds of musicology’s own annals, the far-sighted work of Goehr, Bohlman, Born, and others has drawn full attention to ‘the hegemony of a certain kind of musicology… [which] rests on the ontological assumption that “music’s” core being has nothing to do with the “social” […] such that the appropriate focus in music scholarship is on “the ‘music itself” (Born, 2010, p. 208). Having recognized this essentialism for what it is, scholars have identified its impact on the politics of scholarship and disciplinarity (Bohlman, 1993), spotting also its “preoccupation with the bounded, internal, immanent development of the lineages of Western art music” (Born, 2010, p. 209). Following such public recognition (in the academic sphere, at least) of this tendency to essentialize, and of its legacy on the remit and conceptual vocabulary of wider musicological research, one might expect the problem to be resolved.

However, while the wealth of the English language alone provides many ways to point at very particular musical encounters from the pragmatic (“jam”) to the abstract (“work”) to the esoteric (“harmonic progression”), such identification remains absent too frequently. It is more convenient to use “music” as an all-purpose stand-in for the most readily available objects of musical analysis (“score,” “recording,” “performance”). While such shorthand is understandable – desirable, even – within well-defined disciplinary communities, it can become problematic when researchers from many different disciplinary backgrounds reach out to the same topic, as in the burgeoning popularity of current alignments in music and science. Across cognitive science, psychology and neuroscience, for example, there is a huge risk of carrying over that same “ontological assumption” that Bohlman identified 20 years ago: that a single, essentialized concept of Western classical “music” may stand alone as an object of analysis (Cross, 2003). Given that the research program loosely identified as embodied music cognition represents just such interdisciplinarity, this is an issue worth serious consideration.

### INDIVIDUALISM

At the outset of this paper, I proposed that an enduring problem of “individualism” limits the ultimate power of social explanations of musical behavior. This problem is not obvious; in fact, it is difficult to notice for its very pervasiveness. Following the popularity of abstract cognitivism in the mid- to late-20th century, the resulting lack of attention to the role of physical bodies in experience and cognition had become increasingly obvious by the 1990s. In music, researchers such as Jane Davidson took advantage of newly accessible technologies of video recording and multimedia data management to focus on the analysis of performers’ body movement (Davidson, 1993). There has since been a widespread acknowledgment that musicians move in various ways that exceed the demands of sound production on their instruments (Wanderley and Vines, 2006; Desmet et al., 2012); and an increasing understanding of the way that individuals respond to music through movement (Keller and Rieger, 2009; Toiviainen and Keller, 2010; Toiviainen et al., 2010; Burger et al., 2012).

Methodologically, it seems that an embodied agenda should be equipped to tackle all sorts of different “musical encounters,” and be able to bring under analytical scrutiny the actual event of musical performance in any domain: performers with performers, performers with listeners, classical Western music, “world music,” popular music, sacred music, music from concert halls, music from gigs, music from the streets. Indeed, research is flourishing with diverse musical situations appearing as the subject matter of empirical studies, including flamenco performance (Maduell and Wing, 2007), South African singing (Himberg and Thompson, 2011), and Chinese Guqin performance (Leman et al., 2009), alongside the examples already cited of North Indian classical music performance (Rahaim, 2012; Moran, 2013a,b) and popular music (Davidson, 2001, 2006). There exists a wide range of original research with several significant strands, including work on the varied functions of performer gestures; on the effects of auditory and visual limits on performers’ synchronisation abilities; on the extent of audio-visual interaction in perception of a performance; on the effect of a performer’s expressive movements on an audience’s response to a performance, and so on. Entering the combined terms “*music*^∗^,” “*movement*,*”* and “*perform*^∗^” into the search engine, Google Scholar, in March 2013, and filtering for original, empirical studies of musician communication, a summary literature review retrieved 86 relevant abstracts representing work from sub-fields including systematic musicology, music psychology, and performance research, and offering a corpus that puts physicality center stage in the study of human musicality. However, examination of those 86 abstracts is revealing: in terms of the type of musical material used as a component of the method, 65 studies use scored, notated examples representing classical Western European conventions, compared to just 21 which deal with all and any other non-notated forms of music.

In order to assess the current state of knowledge, and to progress understanding of human musical performance, it is important to draw together evidence across the corpus. But the abstracts of those studies predicated in some way on classical music conventions of notation rarely specified this focus: do the authors presume performance to be the manifestation of a “work,” or an event of social interaction? Is performance an act of social belonging, or of individual expression? Is solo performance the same object of study as ensemble performance? What function is being presumed for musical performance? What do we think it is for? To whom do we think it is directed? Across the board, such theoretical foundations are indistinct. Aside from the relatively small field of cross-cultural music psychology (Stevens, 2012), the majority of significant, contemporary scientific publications in music cognition focus on the conventions of one specific tradition. These studies frame their research question around an implicit focus on notated, Western musical forms, which are ontologically founded on the evocation of an extant, narratively constructed work that is typically based on harmonic progression, and which is conceived as something largely analogous to its notated manifestation. This means that even where a decision to focus on, say, classical string quartet performance, is explicit, the impact of particular ontological categories inherent in that form of music-making tend not to be made explicit.

The following examples are drawn from some important publications (*my emphasis*):

•“…the majority of studies have focused on how listeners perceive emotions expressed *in the music*…” (Juslin and Västfjäll, 2008, p. 561)
•“…the present study investigated simultaneous processing of language and music… [*using] “musically syntactic regular and irregular chord functions*…” (Koelsch et al., 2005, p. 1565).
•“We begin this paper with two fundamental questions about musical representations in humans: first, to what extent are listeners sensitive to *the expressive intent of composers*…?” (Chapados and Levitin, 2008, p. 640).
•“We sought to preserve ecological validity by using a piece of music in *the standard repertoire* by *a major composer*, and recordings of live performances as stimuli.” (Vines et al., 2011, p. 159).

Of course, this small selection does not accurately represent the full breadth and depth of all empirical music performance research. Nonetheless, there is a notable absence in this particular literature of any alternative theoretical basis for construing musical meaning and performance function that is clearly articulated as a matter of study design, and deemed important enough to feature in scientific abstracts. Recall that this field inherited assumptions that were nurtured within cognitivism, studying the perception of acoustic signals and their structures according to compositional features that are the historical subjects of classical Western music analysis. “Music,” as the subject of scrutiny, is all too often an essentialized concept based on the listener-versus-“work” encounter, behind which lies a highly individualistic notion of musical conception, musical performance, and musical listening, based on the Western European classical art tradition.

### MUSICAL COMMUNICATION

Issues of communication unite various threads arising in embodied music research. Typical questions pursued by the 86 abstracts deal with topics in musical communication, for example: Under what conditions of audio-visual communication can co-performers achieve synchronized actions? Which categorical descriptions of emotional state can be communicated within the context of music performance? What type of information is communicated through specific co-performers’ gestures? The notion that music communicates something is undisputed. The key question, then, is: according to what framework – what theorized musical experience – do we examine this event of communication and its underlying cognitive architecture?

Kendall and Carterette’s (1990, p. 131) influential paper, “The Communication of Musical Expression,” proposes a communicative process that involves “an intended musical message, recoded from ideation to notation by the composer, then recoded from notation to acoustical signal by a performer, and finally recoded from acoustical signal to ideation by the listener.” There is no veiled issue of ethnocentrism, since the authors are explicit about the limitations of their approach: “We deal only with traditional Western art music in which composer, performer, and listener are involved” (Kendall and Carterette, 1990, p. 131, footnote 1).

Jones and Holleran (1992) expand on the concept using the particular terms of information theory: “Communication, virtually by definition, assumes a low level of uncertainty with respect to some shared idea of speaker and listener or, in the case of music, of performer/composer and listener” (Jones and Holleran, 1992, p. 4). This transparent presentation defines musical communication as an event of shared meaning, for which the analogy between speaker and performer/composer versus listener can transpose to multiple events of musical encounter.

Compare this to a subsequent, influential publication: “Composers code musical ideas in notation, performers recode from the notation to acoustical signal, and listeners recode from the acoustical signal to ideas. Each performer has intentions to convey; the communicative content in music performance includes the performers’ conceptual interpretation of the musical composition” (Palmer, 1997, pp. 118–119). Palmer (1997) moves beyond Kendall and Carterette’s (1990) more simplistic information-transmission model to include the act of performance itself, pointing out that the individual contribution of the performer does not feature in theoretic music analyses and that this is an area to which empirical research can significantly contribute. She states that “there is no single ideal interpretation for a given musical piece” (Palmer, 1997, p. 119).

**FIGURE 2:**
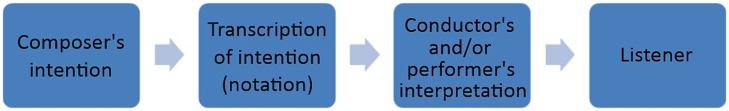
**Linear information-transmission model of musical communication.** In this model, a performer evokes the work and delivers it to the listener. The work is conceived of as a fixed entity. The intentions are contained in the work, to be channeled by the performer. The performance exists to serve the work.

Notice that by acknowledging that multiple “interpretations” exist, it must be taken for granted that – while modulated to some degree by the nuance of individual performance – musical communication can be reduced to the listener’s encounter with a true “work.” This conception of music requires the analytical separation of acts of composition, interpretation, and performance, and assumes a distinct audience of non-participatory listeners. Palmer’s sophisticated model is subtle, but it is essentially conceived as a linear “intention–notation–interpretation–reception” chain (see **Figure 2**). This model of musical meaning – from composer via (conductor via) performer to audience – happens to fit neatly with the prevailing listener-versus-“work” idea of what a musical encounter entails. However, it is problematic for at least three reasons. Firstly, it is clearly an incomplete account of a complex social phenomenon (Hargreaves et al., 2005). Secondly, its essentialization reduces all musical behavior to a time-neutral phenomenon based on a European art-history perspective. While this may be appropriate for an explication of a musical “work,” music in performance is a process and it has duration. Finally, based as the model is on instantiated, scriptable intentions, it cannot access those aspects of musical meaning which are not served by language, but which arise instead through the social interaction processes at the heart of live performance. The crux of the linear transmission account is the notion of an idealized and external musical “work.” This is tied up with the theory and practice of notation; with the culture of a particular art form that values individual acts of composition; and sits within a technological and economic practice which separates the concepts of composition, interpretation (often by an individual conductor), and performance (often by individual professional performers). Listener-versus-“work” is not the only musical encounter that deserves explanation, but its hegemonic status commands a disproportionate amount of attention.

Returning to the three, mid-1990s accounts of musical communication, Jones and Holleran’s information theory model has a particular strength in that it does not actually specify one-way transmission. The idea of communication evoked here includes an act – an event – of sharing. It does not directly implicate an abstract musical “work.” Its analogy to speakers and listeners can be interpreted as a nod to the spontaneous, interactive aspects of much human musical behavior. It thus provides some space to consider the event of musicking: the emergent performance. It also demands some specific propositional agreement – a “low level of uncertainty with respect to some shared idea” (Jones and Holleran, 1992, p. 4). Considering the 20th-century development of cognitive science alongside computational linguistics, the analogy of music and language is almost irresistible (Clarke, 1989) – and a characteristic assumption of cognitivist music psychology is that the “shared idea” will typically imply semantic content, that being a particular attraction for those interdisciplinary researchers who address music via the specific theoretic framework of Western European art tradition. In cross-cultural perspective, this particular musical ecology has a highly developed system of musical literacy that involves an unusually prescriptive notation. Therefore it seems natural to search out some propositional aspect equivalent to that found in words and language – and indeed, irrefutable evidence points to the overlap between our cognitive experience of musical and linguistic forms (Mithen, 2005; Patel, 2010). Since considerable scientific efforts on the topic of music and language have significantly progressed our understanding of brain and behavior, it would be entirely misguided to disregard this work. Regardless, it is no less important to acknowledge biases in the way that music research agendas have often been set: according to the characteristics of a particular type of musical encounter that happens to be bound up with scored, notated forms of dissemination. Most musical ecologies do not have the same relationship with notation, including musical interaction between carers and infants, improvised music therapy, child-led playground music, work songs, ritual, popular music, traditional (folk) music forms, classical Asian art musics, and so on. In the face of those alternative musical experiences described above, the essentialized musical “work” encounter at the heart of most analytical and theoretical approaches seems to fade away.

Music scholarship is manifestly not a natural science. “Music” is as dynamic and complex a phenomenon as the “society” that enacts it, and suited to the same detailed, philosophic scholarship. But as a universal human practice, it also deserves and requires serious evidence-based, systematic investigation. All such research is inherently interdisciplinary, as it must start from ontological assumptions about the nature of the musical behavior at the heart of the enquiry: such assumptions derive from a musicological basis, not a value-free, universally truthful account of the purpose and organization of human musical behavior. The ultimate aim of scientific research is to offer explanation with as much power and certainty as possible and therefore – rightly or wrongly – public appraisal tends to be biased, assuming a scope of explanation with universal (rather than specific) implications. Thus, scientific accounts of cognition that are reliant on culturally specific and yet under-specified musical encounters are of particular concern. By its very design, scientific enquiry requires conscious and significant reduction of the object of analysis on the part of the investigator. It seems unlikely, then, that there will be one single solution to the problem of how to reduce the complex dimensions of “music” into a tractable object. More useful and practicable would be an inclusive account of musical performance as the basic premise of interdisciplinary research (a recent review by Phillips-Silver and Keller (2012) provides a valuable discussion on this point).

I have suggested that a linear transmission model is implicit in a great deal of research, and that this particular model’s object of enquiry deals with music as an individualistic and abstract encounter. However, the field of music performance research has advanced considerably in scale and scope in the past few years and recently social interaction in music has received greater attention. Researchers more frequently address ensemble performance, concurrently devising new technical methods of data capture and analysis. Palmer (2013) and Repp and Su (2013) provide the most thorough reviews of performer movement studies and sensorimotor synchronization studies, respectively. Nonetheless, the majority of the studies reported in these reviews deal with music based in a Western classical tradition, and for various reasons they approach ensemble performance from the perspective of separable, individual behaviors rather than by exploring the constitutive role of the interaction process itself.

The fundamental importance of interaction in the study of music has been emphasized most categorically by Marc Leman’s extensive and continuing collaborative research (Leman, 2008). Various interdisciplinary research programs have also directly addressed social interaction (for example, EmcoMetecca (Leman, 2008), and PESI (Correia et al., 2013)). Beyond initial theoretical proposals (Leman, 2007), one could note that the key contribution of this important and productive body of work lies in empirical observations and technical method development, rather than substantial revision of the ontological premises of the musical encounter.

But what should an alternative model of musical communication represent? Empirical research dealing with joint musical action and behavior has flourished in the past few years, and is now beginning to address more nuanced social qualities than those related to purely rhythmical synchronization (Repp and Su, 2013). In contrast to an information-transaction view of social interaction, the embodied perspective of face-to-face communication (Watzlawick et al., 1967; Cassell et al., 2000) immediately highlights the co-operative, emergent aspects implicated in joint activities like music making. Attention to these micro-social dimensions of musical behavior and performance opens the door to the rich context of all acts of human interaction, primarily the non-verbal elements such as timing, gesture, and utterance.

### MUSICAL INTERACTION, MUSICAL ENACTION, AND KISSING

Finally, this is the moment to compare music with kissing. Take the opportunity to think about a kiss. Not a peck on the cheek, but a nice, long kiss. One person alone cannot do this kiss: they cannot even do half of it, because the kiss only exists in performance. Its subtleties of movement and response require joint behavior. Consider also how your imagined kiss is not instantaneous; a good one will take time. It is certainly an act of face-to-face communication, and words (for more reason than one) have nothing to do with it. Kissing is play. It can be serious, but in experience it is playful in its non-goal-orientedness, its emergence, and its lack of implication for the essential chores of everyday life.

I raise this analogy with serious intent, to make two specific points: (1). There are ways of behaving communicatively which are simply not meaningfully reducible to the separate actions of two individuals. (2). While kissing (and the rest) is perhaps the ultimate act of face-to-face interaction, all spontaneous, non-scripted social interaction takes this co-performed pattern. It is unlikely that empirical approaches which are implicitly biased toward an individual conception of music-making will be able to explain well the sort of direct, non-linguistic, co-constructed meaning which – it is argued – is emergent from the interactional behavior of musicking people.

**FIGURE 3:**
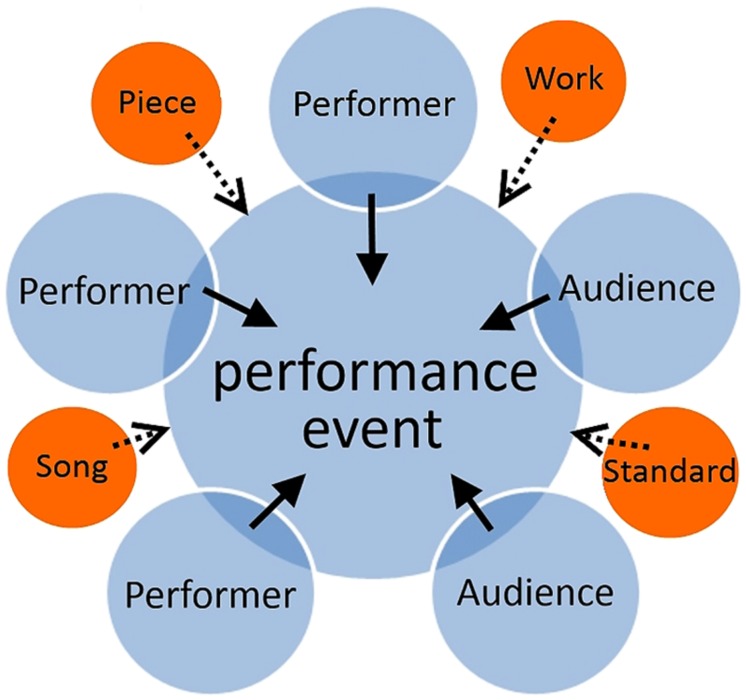
**Emergent-Interactional model of musical communication.** Performers enact a performance event, which could be founded on a piece, song, standard, or work. Listeners attend to the performance. The performance is emergent from the actions of all the participants. Where a “work” is being enacted, this exists to serve the performance.

Born (2010, p. 232) articulates the reductive dangers of suggesting that music mediates solely via the social context of performance. And yet, for interdisciplinary music research directed to a science-based rather than a humanities-based audience, the greater danger may be to assume that this social context is included in the research topic when in fact it is not. The alternative, emergent model of musical communication proposed in **Figure 3** places the performance event as the central concern. In this model, while performers, ideas, and audiences are required to make the musical performance happen, if the musical performance does not happen then there is no object of analysis. This basic conception of musical communication thus offers an ontologically grounded starting point for the empirical study of face-to-face musical encounters, recognising that an event of music-making is generated in the process of live performance.

Returning once more to the notion of musical encounters, an empirical focus on the performance of music-as-play may offer something more social and interactive than can music-as-work or music-as-piece, a key difference being the foregrounding of process. Some recent research supports the notion that the aspect of interpersonal relationship is a vital constituent of co-performed (especially non-scripted or improvised) music, and that this manifests as a highly reciprocal interaction process. Duffy and Healey (2013), for example, describe how musical contributions during a practical music lesson are closely integrated with other verbal and non-verbal cues as part of the unfolding conversation, while Moran (2013b) reports on the constitutive function of musicians’ expressive gestures in North Indian co-improvisation. Empirical methods are therefore required to make the musical interaction itself into the object of analysis; and indeed, various innovative research now attends to this issue. There is growing theoretical support for such an enactivist approach – for example, De Jaegher and Di Paolo’s (2007) work on Participatory Sense-Making argues that “the interaction process can take on a form of autonomy. This allows us to reframe the problem of social cognition as that of how meaning is generated and transformed in the interplay between the unfolding interaction process and the indiviuals engaged in it” (De Jaegher and Di Paolo, 2007, p. 485). Importantly, this is not an argument that musical “meaning” can be reduced to the physical components of performance process; rather that a proposed autonomous interaction process itself may be a reckonable dimension in the experience of live (ensemble) music performance.

In extant music research, the growing body of sensorimotor synchronization studies as led and reviewed by Bruno Repp (Repp, 2005; Repp and Su, 2013), and the work of Peter Keller (2014) and his collaborators is of prime importance from a systems perspective. Palmer’s (2013) review draws together evidence from movement and timing analyses by Goebl and Palmer (2009) and Konvalinka et al. (2010), Loehr and Palmer (2011), to report that duo synchronization appears to arise from mutual adaptation which “transcend[s] influences of musical roles and any biomechanical differences” (Palmer, 2013, p. 416); while Badino et al. (2014) report that the anticipation of others’ action is “a necessary prerequisite for successful joint action control” given the complexity of musical ensemble behavior, and the instantaneous nature of interpersonal response (Badino et al., 2014, p. 102). Using variations on interactive paradigms to explore interpersonal interaction in music-related settings, the studies all contribute evidence that orients us toward a better understanding of the physical manifestation of “unity” by individuals involved in a joint activity. Typically, such research is framed in terms of identifying the cognitive architecture behind motor-related joint synchronization problems, and is largely (though not exclusively) focused on synchronization via the auditory modality.

Much important work on interaction has been carried out through the psychological study of “joint action” (Knoblich et al., 2011); however, some researchers have devised paradigms that address the particular role of interaction processes to social aspects of cognition: Phillips-Silver and Trainor (2005), Hove and Risen (2009), Kirschner and Tomasello (2009, 2010), Launay et al. (2013), and Rabinowitch et al. (2013) are notable for music-specific research. Two key precursors to this distinct methodological stance – originally devised for non-music interaction research – are also worth describing in order to demonstrate the pioneering shift in their attention toward the interaction process itself. Firstly, the “double video” paradigm devised by Murray and Trevarthen (1985) for research on infant-carer responsiveness. This original paradigm inspired many replications and variations. Murray and Trevarthen (1985) used a closed television circuit to control conditions of face-to-face interaction between four infant-carer dyads. The four 2 month-old infants were seen to look significantly less at their mother during a televised replay of the mother’s behavior compared to when they engaged in televised live interaction. The study and subsequent variations (Nadel et al., 1999; Stormark and Braarud, 2004) have demonstrated the socially contingent and interactive nature of the facial expressions and vocalizations used by both parties in the dyad. The second example comes from Auvray et al.’s (2009) “perceptual crossing” design. This experiment examined the extent to which the mechanisms underlying the perception of mutual participation in a shared activity are contingent upon features of the shared activity itself. In a virtual environment, participants in the study were able to discriminate between sensory (haptic, tactile) feedback which they received when their virtual agent encountered (“crossed”) the avatar of another person. They could discern the other agent’s presence from the feedback that they received via interaction with either a static or a mobile inanimate “lure.” The authors demonstrated that the interdependence of their perceptual activities underlies participants’ strategies for correct identification of another agent. In other words, the distinguishing property of mutual perception (rather than other-object-perception) is one that is emergent from the joint activities of the two participants. As it is contingent upon their real-time interaction, this aspect of social cognition is demonstrably non-reducible to the individual mind.

Focusing on the process of social interaction itself, evidence from empirical studies converges on the idea that attentiveness and/or agency-revealing cues between social interactors are mutually contingent: behavioral cues depend upon and also constitute the meaningful events that arise through the interaction context. Such evidence has lent support to increasingly radical embodiment agendas (Marsh et al., 2009; Di Paolo et al., 2010) which address the specific analytical object of the autonomous interaction process. Similar evidence for an emergent, autonomous interaction process in co-performed music may provide a specific and tractable problem for empirical music research, offering a particular way of approaching the social, embodied experience of live music performance. Recent work on such lines is underway: output from the SIEMPRE project directly addresses the emergent aspects of social interaction in co-performance, including a study examining whether observers can judge from a recording of a violinist whether he is playing alone or in a group (Glowinski et al., 2013). A further study explored the impact on a sensorimotor co-ordination between string quartet members of the first-violinist’s communication of previously undiscussed temporal and dynamic changes (Badino et al., 2014). Other work has begun to explore the neural bases of musical interaction; focusing on the behavior of ensemble performers, Lindenberger et al. (2009) used dual EEG to study within-brain synchronization by duetting guitarists; and Keller and his collaborators have continued to develop the “adaptive partner” paradigm within fMRI designs (Fairhurst et al., 2013).

## CONCLUSION

This paper set out to examine the social implications of an embodied approach to music cognition, proposing that the prevailing understanding of musical communication, underpinning a broad range of research into perception and performance, may yet rest on an individualistic account of a socially oriented human behavior. Based on the musical ecology of score-based, notated art music – the dominant object of analysis for most interdisciplinary music research – the unacknowledged use of an essentially linear model of musical communication may be capping the potential and influence of embodied accounts of music cognition. I have described some alternative examples of current empirical music studies which sit firmly within a general trend of embodied music cognition research, but which push forward an “interaction” strand within it. This enactivist perspective appears to offer great potential for the study of genuinely interactive, social aspects of human music making, and is yielding innovative research designs, and data analysis methods.

The social implications of research into embodied music cognition are extensive and important, sitting behind those issues of process and emergence that are central to all lived, experienced musical encounters – including the case of dance, a topic which has brought out similar scholarly concerns (Reynolds and Reason, 2012). Having acknowledged the central role of bodies and human movement to cognition, we must not assume that the concomitant matters of social context and the performance of our social relationships are taken care of. Compromised by underlying presumptions about the nature of musical experience, a partial acknowledgment of the problem of ethnocentricity not only restricts research findings, but also serves a continuing political division between “valid” music scholarship (addressing analysis of “the music,” meaning art music), versus “soft” music scholarship (addressing “peripheral” social context, encompassing the study of all and any form of musicking not ontologically predicated on literacy). Instead, an active focus on those interaction processes that appear to co-constitute embodied music cognition may offer the best opportunity to examine something vitally important: that music is a social phenomenon, enacted as meaningful, social encounters through playful interaction.

## Conflict of Interest Statement

The author declares that the research was conducted in the absence of any commercial or financial relationships that could be construed as a potential conflict of interest.

## References

[B1] Anderson M. L. (2003). Embodied cognition: a field guide. *Artif. Intell.* 149 91–130 10.1016/S0004-3702(03)00054-7

[B2] Auvray M. Lenay C. Stewart J. (2009). Perceptual interactions in a minimalist virtual environment. *New Ideas Psychol.* 27 32–47 10.1016/j.newideapsych.2007.12.002

[B3] Badino L. D’ausilio A. Glowinski D. Camurri A. Fadiga L. (2014). Sensorimotor communication in professional quartets. *Neuropsychologia* 55 98–104 10.1016/j.neuropsychologia.2013.11.012 24333167

[B4] Barsalou L. W. (2008). Grounded cognition. *Annu. Rev. Psychol.* 59 617–645 10.1146/annurev.psych.59.103006.093639 17705682

[B5] Becker J. O. (2004). *Deep Listeners: Music, Emotion, and Trancing*. Bloomington, IN: Indiana University Press

[B6] Bharucha J. Krumhansl C. L. (1983). The representation of harmonic structure in music: hierarchies of stability as a function of context. *Cognition* 13 63–102 10.1016/0010-0277(83)90003-3 6681743

[B7] Bohlman P. V. (1993). Musicology as a political act. *J. Musicol.* 11 411–436 10.2307/764020

[B8] Boone R. T. Cunningham J. G. (2001). Children’s expression of emotional meaning in music through expressive body movement. *J Nonverbal Behav.* 25 21–41 10.1023/A:1006733123708

[B9] Born G. (2010). For a relational musicology: music and interdisciplinarity, beyond the practice turn. *J. R. Music. Assoc.* 135 205–243 10.1080/02690403.2010.506265

[B10] Bowman W. (2004). “Cognition and the body: perspectives from music education,” in *Knowing Bodies, Moving Minds: Towards Embodied Teaching and Learning* ed. Bresler L. (Netherlands: Kluwer Academic Press), 29–50

[B11] Broughton M. Stevens C. (2009). Music, movement and marimba: an investigation of the role of movement and gesture in communicating musical expression to an audience. *Psychol. Music* 37 137–153 10.1177/0305735608094511

[B12] Burger B. Thompson M. R. Luck G. Saarikallio S. Toiviainen P. (2012). “Music moves us: beat-related musical features influence regularity of music-induced movement,” in *The 12th International Conference on Music Perception and Cognition and the 8th Triennial Conference of the European Society for the Cognitive Sciences of Music* eds Cambouropoulos E. Tsougras C. Mavromatis P. Pastiadis K. (Thessaloniki: ICMPC-ESCOM).

[B13] Cadoz C. Wanderley M. M. (2000). “Gesture-music,” in *Trends in Gestural Control of Music* eds Wanderley M. M. Battier M. (Paris: IRCAM), 1–55

[B14] Camurri A. Hashimoto S. Ricchetti M. Ricci A. Suzuki K. Trocca R. (2000). Eyesweb: toward gesture and affect recognition in interactive dance and music systems. *Comput. Music J.* 24 57–69 10.1162/014892600559182

[B15] Camurri A. Mazzarino B. Ricchetti M. Timmers R. Volpe G. (2004). “Multimodal analysis of expressive gesture in music and dance performances,” in *Gesture-Based Communication in Human-Computer Interaction* eds Camurri A. Volpe G. (Berlin: Springer), 20–39 10.1007/978-3-540-24598-8_3

[B16] Cassell J. Sullivan J. Prevost S. Churchill E. eds. (2000). *Embodied Conversational Agents*. Cambridge, MA: MIT Press

[B17] Chapados C. Levitin D. J. (2008). Cross-modal interactions in the experience of musical performances: physiological correlates. *Cognition* 108 639–651 10.1016/j.cognition.2008.05.008 18603233

[B18] Clarke E. F. (1989). Issues in language and music. *Contemp. Music Rev.* 4 9–22 10.1080/07494468900640181

[B19] Clarke E. F. (1993). “Generativity, mimesis and the human body in music performance,” in *Music and the Cognitive Sciences* eds Cross I. Deliège. I. (Switzerland: Harwood Academic Press), 207–219

[B20] Clayton M. Sager R. Will U. (2004). In time with the music: the concept of entrainment and its significance for ethnomusicology. *ESEM CounterPoint* 1 1–45

[B21] Cook N. (1994). “Perception: a perspective from music theory,” in *Musical Perceptions* eds Aiello R. Sloboda J. (Oxford: Oxford University Press), 64–95

[B22] Correia N. N. Tahiroǧlu K. Espada M. (2013). “PESI: extending mobile music instruments with social interaction,” in *Seventh International Conference on Tangible, Embedded and Embodied Interaction (TEI)* Barcelona.

[B23] Cox A. (2001). The mimetic hypothesis and embodied musical meaning. *Music. Sci.* 5 195–212 10.1177/102986490100500204

[B24] Cross I. (1998). Music and Science: Three Views. *Revue Belge de Musicologie LII* 207–214 10.2307/3686926

[B25] Cross I. (2003). “Music and biocultural evolution,” in *The Cultural Study of Music: A Critical Introduction* eds Clayton M. Herbert T. Middleton R. (London: Routledge), 19–30

[B26] Davidson J. W. (1993). Visual perception and performance manner in the movements of solo musicians. *Psychol. Music.* 21 103–113 10.1177/030573569302100201

[B27] Davidson J. W. (2001). The role of the body in the production and perception of solo vocal performance: a case study of annie lennox. *Music. Sci.* 5 235–256 10.1177/102986490100500206

[B28] Davidson J. W. (2006). “She’s the One’: multiple functions of body movement in a stage performance by Robbie Williams,” in *Music and Gesture* eds Gritten A. King E. (Aldershot: Ashgate), 208–226

[B29] De Jaegher H. Di Paolo E. (2007). Participatory sense making. *Phenomenol. Cogn. Sci.* 6 485–507 10.1007/s11097-007-9076-9

[B30] Desmet F. Nijs L. Demey M. Lesaffre M. Martens J.-P. Leman M. (2012). Assessing a clarinet player’s performer gestures in relation to locally intended musical targets. *J. New Music Res.* 41 31–48 10.1080/09298215.2011.649769

[B31] Deutsch D. (1999). “Grouping mechanisms in music,” in *Psychology of Music* ed. Deutsch D. (New York, NY: Gulf Professional Publishing), 299–348

[B32] Di Paolo E. A. Rohde M. De Jaegher H. (2010). “Horizons for the enactive mind: values, social interaction, and play,” in *Enaction: Towards a New Paradigm for Cognitive Science* eds Stewart J. Gapenne O. Di Paolo E. A. (Cambridge, MA: MIT Press), 33–87

[B33] Doǧantan-Dack M. (2006). The body behind music: precedents and prospects. *Psychol. Music* 34 449–464 10.1177/0305735606067155

[B34] Duffy S. Healey P. G. T. (2013). “Using music as a turn in conversation in a lesson,” in *Proceedings of the 35th Annual Conference of the Cognitive Science Society.* eds Knauff M. Pauen M. Sebanz N. Wachsmuth I. (Austin, TX: Cognitive Science Society), 2231–2236

[B35] Eitan Z. Granot R. Y. (2006). How music moves: musical parameters and listeners images of motion. *Music Percept.* 23 221–248 10.1525/mp.2006.23.3.221

[B36] Fairhurst M. T. Janata P. Keller P. E. (2013). Being and feeling in sync with an adaptive virtual partner: brain mechanisms underlying dynamic cooperativity. *Cereb. Cortex* 23 2592–2600 10.1093/cercor/bhs243 22892422

[B37] Gallagher S. (2005). *How the Body Shapes the Mind*. Cambridge: Cambridge University Press 10.1093/0199271941.001.0001

[B38] Ginsborg J. Chaffin R. Nicholson G. (2004). “Sharing performance cues in collaborative performance: a case study,” in *ICMPC8* eds Lipscomb S. Ashley R. Gjerdingen R. O. Webster P. (Adelaide: Causal Productions), 252–255

[B39] Glowinski D. Mancini M. Cowie R. Camurri A. Chiorri C. Doherty C. (2013). The movements made by performers in a skilled quartet: a distinctive pattern, and the function that it serves. *Front. Psychol.* 4:841 10.3389/fpsyg.2013.00841 PMC3826428 24312065

[B40] Godøy R. I. Leman M. (eds) (2010). *Musical Gestures: Sound, Movement and Meaning*. New York: Routledge

[B41] Goebl W. Dixon S. De Poli G. Friberg A. Bresin R. Widmer G. (2005). “Sense in expressive music performance: data acquisition, computational studies, and models,” in *Sound to Sense, Sense to Sound: A State-of-the-Art* eds Polotti P. Rocchesso D. (Berlin: Logos Verlag Berlin).

[B42] Goebl W. Palmer C. (2009). Synchronization of timing and motion among performing musicians. *Music Percept.* 26 427–438 10.1525/mp.2009.26.5.427

[B43] Goehr L. (1992). *The Imaginary Museum of Musical Works: An Essay in the Philosophy of Music*. Oxford: Oxford University Press

[B44] Gritten A. King E. eds. (2006). *Music and Gesture*. Aldershot: Ashgate

[B45] Gritten A. King E. (2011). *New Perspectives on Music and Gesture*. Aldershot: Ashgate

[B46] Hargreaves D. J. Macdonald R. Miell D. E. (2005). “How do people communicate using music?,” in *Musical Communication* eds Miell D. E. Macdonald R. Hargreaves D. J. (Oxford: Oxford University Press), 1–19

[B47] Himberg T. Thompson M. R. (2011). Learning and synchronising dance movements in South African songs - a cross-cultural motion-capture study. *Dance Res.* 29 305–328 10.3366/drs.2011.0022

[B48] Hove M. J. Risen J. L. (2009). It’s all in the timing: interpersonal synchrony increases affiliation. *Soc. Cogn.* 27 949–960 10.1521/soco.2009.27.6.949

[B49] Iyer V. (2002). Embodied mind, situated cognition, and expressive microtiming in African-American music. *Music Percept.* 19 387–414 10.1525/mp.2002.19.3.387

[B50] Jones M. R. Holleran S. (1992). *Cognitive Bases of Musical Communication: An Overview*. Washington, DC: American Psychological Association 10.1037/10104-000

[B51] Juslin P. N. Timmers R. (2010). “Expression and communication of emotion in music performance,” in *Handbook of Music and Emotion: Theory, Research, Applications* eds Juslin P. N. Sloboda J. A. (New York: Oxford University Press), 453–489

[B52] Juslin P. N. Västfjäll D. (2008). Emotional responses to music: the need to consider underlying mechanisms. *Behav. Brain Sci.* 31:559 10.1017/S0140525X08005293 18826699

[B53] Kawase S. (2014). Gazing behavior and coordination during piano duo performance. *Atten. Percept. Psychophys.* 76 527–540 10.3758/s13414-013-0568-0 24170378

[B54] Keller P. E. (2014). “Ensemble performance: interpersonal alignment of musical expression,” in *Expressiveness in Music Performance: Empirical Approaches Across Styles and Cultures* eds Fabian D. Timmers R. Schubert E. (Oxford: Oxford University Press), 260–282

[B55] Keller P. E. Appel M. (2010). Individual differences, auditory imagery, and the coordination of body movements and sounds in musical ensembles. *Music Percept.* 28 27–46 10.1525/mp.2010.28.1.27

[B56] Keller P. E. Rieger M. (2009). Editorial: special issue – musical movement and synchronization. *Music Percept.* 26 397–400 10.1525/mp.2009.26.5.397

[B57] Kendall R. A. Carterette E. C. (1990). The communication of musical expression. *Music Percept.* 8 129–163 10.2307/40285493

[B58] Kirschner S. Tomasello M. (2009). Joint drumming: social context facilitates synchronization in preschool children. *J. Exp. Child Psychol.* 102 299–314 10.1016/j.jecp.2008.07.005 18789454

[B59] Kirschner S. Tomasello M. (2010). Joint music making promotes prosocial behavior in 4-year-old children. *Evol. Human Behav.* 31 354–364 10.1016/j.evolhumbehav.2010.04.004

[B60] Knoblich G. Butterfill S. Sebanz N. (2011). “Psychological research on joint action: theory and data,” in *The Psychology of Learning and Motivation* ed. Ross B. (Burlington: Academic Press), 59–101

[B61] Koelsch S. Gunter T. C. Wittfoth M. Sammler D. (2005). Interaction between syntax processing in language and in music: an ERP study. *J. Cogn. Neurosci.* 17 1565–1577 10.1162/089892905774597290 16269097

[B62] Konvalinka I. Vuust P. Roepstorff A. Frith C. D. (2010). Follow you, follow me: continuous mutual prediction and adaptation in joint tapping. *Q. J. Exp. Psychol.* 63 2220–2230 10.1080/17470218.2010.497843 20694920

[B63] Krumhansl C. L. (1995). Music psychology and music theory: problems and prospects. *Music Theory Spectr.* 17 53–80 10.2307/745764

[B64] Launay J. Dean R. T. Bailes F. (2013). Synchronization can influence trust following virtual interaction. *Exp. Psychol.* 60:53 10.1027/1618-3169/a000173 22935329

[B65] Leman M. (2007). *Embodied Music Cognition and Mediation Technology*. Cambridge, MA: MIT Press

[B66] Leman M. (2008). *EmcoMetecca Project Description.* Ghent: IPEM, Ghent University. Available at: http://www.ipem.ugent.be/?q=EmcoMetecca [Accessed 21 April, 2014]

[B67] Leman M. Desmet F. Styns F. Van Noorden L. Moelants D. (2009). Sharing musical expression through embodied listening: a case study based on Chinese Guqin music. *Music Percept.* 26 263–278 10.1525/Mp.2009.26.3.263

[B68] Lerdahl F. Jackendoff R. (1983). *A Generative Theory of Tonal Music*. Cambridge, Mass: MIT Press

[B69] Lindenberger U. Li S.-C. Gruber W. Müller V. (2009). Brains swinging in concert: cortical phase synchronization while playing guitar. *BMC Neurosci.* 10:22 10.1186/1471-2202-10-22 PMC2662862 19292892

[B70] Loehr J. D. Palmer C. (2011). Temporal coordination between performing musicians. *Q. J. Exp. Psychol.* 64 2153–2167 10.1080/17470218.2011.603427 21929475

[B71] Maduell M. Wing A. (2007). The dynamics of ensmble: the case for flamenco. *Psychol. Music* 35 591–627 10.1177/0305735607076446

[B72] Marsh K. L. Johnston L. Richardson M. J. Schmidt R. (2009). Toward a radically embodied, embedded social psychology. *Eur. J. Soc. Psychol.* 39 1217–1225 10.1002/ejsp.666

[B73] Mithen S. (2005). *The Singing Neanderthals: The Origins of Music, Language, Mind and Body*. London: Widenfeld & Nicholson

[B74] Moran N. (2013a). Music, bodies and relationships: an ethnographic contribution to embodied cognition studies. *Psychol. Music* 41 5–17 10.1177/0305735611400174

[B75] Moran N. (2013b). “Social co-regulation and communication in North Indian duo performances,” in *Experience and Meaning in Music Performance* eds Clayton M. Dueck B. Leante L. (New York, NY: Oxford University Press), 40–61 10.1093/acprof:oso/9780199811328.003.0003

[B76] Murray L. Trevarthen C. (1985). “Emotional regulation of interactions between two-month-olds and their mothers,” in *Social Perception in Infants* eds Field T. M. Fox N. A. (Norwood: Ablex), 177–197

[B77] Nadel J. Carchon I. Kervella C. Marcelli D. Réserbat-Plantey D. (1999). Expectancies for social contingency in 2-month-olds. *Dev. Sci.* 2 164–173 10.1111/1467-7687.00065

[B78] Palmer C. (1997). Music performance. *Annu. Rev. Psychol.* 48 115–138 10.1146/annurev.psych.48.1.115 9046557

[B79] Palmer C. (2013). “Music performance: movement and coordination,” in *The Psychology of Music* ed. Deutsch D. 3rd Edn (San Diego, CA: Academic Press/Elsevier), 405–422

[B80] Patel A. D. (2010). *Music, Language, and the Brain*. New York: Oxford University Press

[B81] Phillips-Silver J. Keller P. (2012). Searching for roots of entrainment and joint action in early musical interactions. *Front. Human Neurosci.* 6:26 10.3389/fnhum.2012.00026 PMC3288575 22375113

[B82] Phillips-Silver J. Trainor L. J. (2005). Feeling the beat: movement influences infant rhythm perception. *Science* 308:1430 10.1126/science.1110922 15933193

[B83] Rabinowitch T.-C. Cross I. Burnard P. (2013). Long-term musical group interaction has a positive influence on empathy in children. *Psychol. Music* 41 484–498 10.1177/0305735612440609

[B84] Rahaim M. (2012). *Musicking Bodies: Gesture and Voice in Hindustani Music. MIddletown*. Connecticut: Wesleyan University Press

[B85] Repp B. H. (2005). Sensorimotor synchronization: a review of the tapping literature. *Psychon. Bull. Rev.* 12 969–992 10.3758/BF03206433 16615317

[B86] Repp B. H. Su Y.-H. (2013). Sensorimotor synchronization: a review of recent research (2006–2012). *Psychon. Bull. Rev.* 20 403–452 10.3758/s13423-012-0371-2 23397235

[B87] Reybrouck M. (2005). Body, mind and music: musical semantics between experiential cognition and cognitive economy. *Trans. Music Rev.* 9. Available at: http://www.sibetrans.com/trans/articulo/180/body-mind-and-music-musical-semantics-between-experiential-cognition-and-cognitive-economy

[B88] Reynolds D. Reason M. (2012). *Kinesthetic Empathy in Creative and Cultural Practices*. Bristol: Intellect Books

[B89] Shepard R. N. (1982). Geometrical approximations to the structure of musical pitch. *Psychol. Rev.* 89 305 10.1037/0033-295X.89.4.305 7134331

[B90] Stevens C. J. (2012). Music perception and cognition: a review of recent cross-cultural research. *Top. Cogn. Sci.* 4 653–667 10.1111/j.1756-8765.2012.01215.x 22811369

[B91] Stormark K. M. Braarud H. C. (2004). Infants’ sensitivity to social contingency: a “double video” study of face-to-face communication between 2-and 4-month-olds and their mothers. *Infant Behav. Dev.* 27 195–203 10.1016/j.infbeh.2003.09.004

[B92] Timmers R. (2007). Communication of (e)motion through performance: two case studies. *Orbis Music.* 14 116–140

[B93] Toiviainen P. Keller P. E. (2010). Special issue: spatiotemporal music cognition. *Music Percept.* 28 1–2 10.1525/mp.2010.28.1.1

[B94] Toiviainen P. Luck G. Thompson M. R. (2010). Embodied meter: hierarchical eigenmodes in music-induced movement. *Music Percept.* 28 59–70 10.1525/mp.2010.28.1.59

[B95] Varela F. Thompson E. Rosch E. (1991). *The Embodied Mind: Cognitive Science and Human Experience*. Cambridge, MA: MIT Press

[B96] Vines B. W. Krumhansl C. L. Wanderley M. M. Dalca I. M. Levitin D. J. (2005). Dimensions of emotion in expressive musical performance. *Ann. N. Y. Acad. Sci.* 1060 462–466 10.1196/annals.1360.052 16597804

[B97] Vines B. W. Krumhansl C. L. Wanderley M. M. Dalca I. M. Levitin D. J. (2011). Music to my eyes: cross-modal interactions in the perception of emotions in musical performance. *Cognition* 118 157–170 10.1016/j.cognition.2010.11.010 21146164

[B98] Wanderley M. M. Vines B. W. (2006). “Origins and functions of clarinettists’ ancillary gestures,” in *Music and Gesture* eds Gritten A. King E. (Aldershot: Aldgate), 165–191

[B99] Watzlawick P. Bavelas J. B. Jackson D. D. O’hanlon B. (1967). *Pragmatics of Human Communication: A Study of Interactional Patterns, Pathologies, and Paradoxes*. New York: Norton

[B100] Wilson M. (2002). Six views of embodied cognition. *Psychon. Bull. Rev.* 9 625–636 10.3758/BF03196322 12613670

